# The scenario on the origin of translation in the RNA world: in principle of replication parsimony

**DOI:** 10.1186/1745-6150-5-65

**Published:** 2010-11-27

**Authors:** Wentao Ma

**Affiliations:** 1 College of Life Sciences, Wuhan University, Wuhan 430072, P.R. China

## Abstract

**Background:**

It is now believed that in the origin of life, proteins should have been "invented" in an RNA world. However, due to the complexity of a possible RNA-based proto-translation system, this evolving process seems quite complicated and the associated scenario remains very blurry. Considering that RNA can bind amino acids with specificity, it has been reasonably supposed that initial peptides might have been synthesized on "RNA templates" containing multiple amino acid binding sites. This "Direct RNA Template (DRT)" mechanism is attractive because it should be the simplest mechanism for RNA to synthesize peptides, thus very likely to have been adopted initially in the RNA world. Then, how this mechanism could develop into a proto-translation system mechanism is an interesting problem.

**Presentation of the hypothesis:**

Here an explanation to this problem is shown considering the principle of "replication parsimony" --- genetic information tends to be utilized in a parsimonious way under selection pressure, due to its replication cost (e.g., in the RNA world, nucleotides and ribozymes for RNA replication). Because a DRT would be quite long even for a short peptide, its replication cost would be great. Thus the diversity and the length of functional peptides synthesized by the DRT mechanism would be seriously limited. Adaptors (proto-tRNAs) would arise to allow a DRT's complementary strand (called "C-DRT" here) to direct the synthesis of the same peptide synthesized by the DRT itself. Because the C-DRT is a necessary part in the DRT's replication, fewer turns of the DRT's replication would be needed to synthesize definite copies of the functional peptide, thus saving the replication cost. Acting through adaptors, C-DRTs could transform into much shorter templates (called "proto-mRNAs" here) and substitute the role of DRTs, thus significantly saving the replication cost. A proto-rRNA corresponding to the small subunit rRNA would then emerge to aid the binding of proto-tRNAs and proto-mRNAs, allowing the reduction of base pairs between them (ultimately resulting in the triplet anticodon/codon pair), thus further saving the replication cost. In this context, the replication cost saved would allow the appearance of more and longer functional peptides and, finally, proteins. The hypothesis could be called "DRT-RP" ("RP" for "replication parsimony").

**Testing the hypothesis:**

The scenario described here is open for experimental work at some key scenes, including the compact DRT mechanism, the development of adaptors from aa-aptamers, the synthesis of peptides by proto-tRNAs and proto-mRNAs without the participation of proto-rRNAs, etc. Interestingly, a recent computer simulation study has demonstrated the plausibility of one of the evolving processes driven by replication parsimony in the scenario.

**Implication of the hypothesis:**

An RNA-based proto-translation system could arise gradually from the DRT mechanism according to the principle of "replication parsimony" --- to save the replication cost of RNA templates for functional peptides. A surprising side deduction along the logic of the hypothesis is that complex, biosynthetic amino acids might have entered the genetic code earlier than simple, prebiotic amino acids, which is opposite to the common sense. Overall, the present discussion clarifies the blurry scenario concerning the origin of translation with a major clue, which shows vividly how life could "manage" to exploit potential chemical resources in nature, eventually in an efficient way over evolution.

**Reviewers:**

This article was reviewed by Eugene V. Koonin, Juergen Brosius, and Arcady Mushegian.

## Background

### The problem of the origin of protein synthesis in the RNA world

As it was stated by Crick et al., "the origin of protein synthesis is a notoriously difficult problem" [1]. Dating from the 1960 s [2-7], numerous efforts were devoted to this problem, but a convincing "solution" has not been reached so far. The widely accepted idea of the "RNA world" [8] stated that RNA should have emerged first in the origin of life, acting as both genetic and functional molecules [9]. Thus, the problem was provided with a detailed context, in which it could be rephrased as "how proteins could be invented in the RNA world". However, this does not make the problem much simpler or clearer.

Indeed, proteinacious components involved in the modern translation system, including those in the ribosome, aminoacyl-tRNA synthetases (AARSs), and translation factors, have been either evidenced or implied to be absent originally [10-13]. Thus, the paradox "proteins participated in their own origin" could be evaded. However, an RNA version of the translation system, e.g. one involving proto-tRNAs, proto-mRNAs, proto-rRNAs and perhaps some aminoacyl-tRNA synthetase ribozymes, seems to be still too complicated [14].

As it was pointed out, evolution should be continuous [7], "is myopic" [15], or "has no foresight" [16]. It could not work like an engineer, i.e. construct a complex machine according a blueprint, even if the machine would turn out to be enormously beneficial. Thus, the key problem is how the remote goal could have been reached via intermediate steps, each of which was "within the sight" of evolution. Apparently, there could be many possibilities on these intermediate steps for the emergence of different components in the machine, considering their direct advantages, potential original sources, emerging order, etc. Indeed, a number of hypotheses have been proposed, based on various kinds of evidence or consideration. However, most of them only discussed some of the intermediate steps in detail, leaving other steps unclear or only mentioned in an abstract way. Additionally, they tended to treat those intermediate steps separately, e.g. tRNAs, mRNAs and rRNAs were suggested to emerge due to different reasons, and the whole scenario was usually no better than a "patchwork" (because even a brief review on these hypotheses would be too long, for the sake of a quick start, I will explain these hypotheses later in the part "Implication of the hypothesis"). In the following, I will suggest that there could be a major clue in the scenario, which might have accounted for the emergence of almost all the components of an RNA-based proto-translation system. I will try to envision all the intermediate steps in detail, encompassing this major clue, which, in my opinion, makes the whole scenario much clearer.

### The Direct RNA Template (DRT) hypothesis on the synthesis of the first peptides

The "invention" of DNA in the RNA world seems to be not difficult to understand at the aspect that RNA could be "reverse transcribed" into DNA under the mechanism of template-directed synthesis via base-pairing. "Unfortunately", there is not such a simple mechanism for RNA to synthesize proteins. However, in *in vitro *selection experiments searching for RNA aa-aptamers, it was revealed that RNA could bind an amino acid with significant specificity [17-23]. Then, if an RNA containing multiple aa-binding sites could bring the bound amino acids spatially adjacent by structural folding, a peptide might be able to form. That is to say, RNA might aid the synthesis of peptides in such a way, very similar to the template-directed synthesis.

This mechanism has been suggested in the "Direct RNA Template (DRT)" hypothesis [20,22]. There are at least two attractive points for this suggestion. First, it is now solidly experiment-evidenced that RNA can bind amino acids with specificity. Second, this DRT mechanism should be the simplest mechanism for RNA to synthesize peptides, and thus very likely to have been adopted initially in the RNA world [20]. Then, it would be necessary and interesting to formulate an explanation on how such a simple mechanism could evolve into an RNA-based proto-translation system. The speculation of the DRT hypothesis itself on this problem was an abstract one [20,22], short of a detailed explanation on those intermediate steps. In this paper, I will show that starting from the DRT mechanism, almost all the components of an RNA-based proto-translation system, including proto-tRNAs, proto-mRNAs and (some) proto-rRNAs, could emerge gradually under the principle of "replication parsimony". Or say, the main driving force (selective advantage) for the emergence of an RNA-based proto-translation system should be the "replication parsimony". This should have been the major clue in the scenario on the origin of translation in the RNA world.

Here, by "replication parsimony" I mean that informational sequences in genetic molecules have a tendency to be utilized in a parsimonious way under selection pressure, due to the cost involved in their replication. The principle should be very important at least in early stages of life. For instance, it is well known that there is little non-coding DNA in prokaryote genomes. In some viruses there are even overlapping genes coding for different proteins. In an RNA world, the saving of replication cost should have been particularly important because the synthesis of RNA precursors (i.e. nucleotides) and the replication of RNA would have to be catalyzed by ribozymes [9], which should be less efficient than their proteinacious counterparts in modern organisms. In addition, perhaps also important, the ribozyme-catalyzed RNA-replication should be more error-prone [24], which would bring an apparent limitation on the RNA organisms' "genome" size [25] (though this limitation seems to be not so serious as it was previously deemed [26,27]).

## Presentation of the hypothesis

### Compact direct RNA templates: according to "replication parsimony"

**Figure 1:**
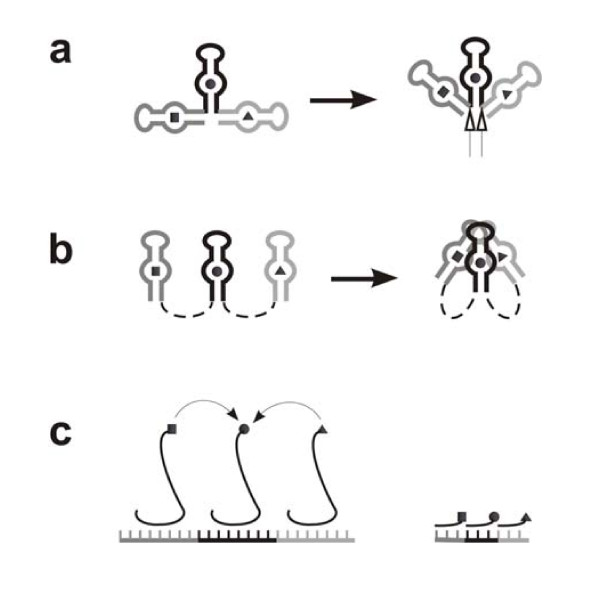
**The stereochemical consideration on the structure of direct RNA templates (DRTs) for peptide synthesis under the principle of replication parsimony**. The dark gray, black and light gray portions of the RNAs correspond to the aa-binding sites for three different amino acids, the square, circle and triangle, respectively. (**a**) If the aa-binding sites in a DRT were organized one after another, stereochemical limitation at their joints (empty arrows) would prevent the folding to bring the amino acids adjacent. Note: it is for convenience to draw the bound amino acid inside the internal loop, with the understanding that the nucleotide residues in other portions of the aptamer domain might also participate in the binding. (**b**) The stereochemical limitation could be overcome by the introduction of spacer sequences (dashed lines) at the joints. (**c**) The stereochemical limitation might also be "compensated" by the spatial stretch of adaptors (S-shape lines, only as an abstract structural model), which recognize the template RNA at one end while charge amino acids at the other end (Left). In the CCH hypothesis [30], the spatial stretch of the supposed adaptors, each composed of a nucleotide triplet (anticodon) charged with an amino acid, is limited (Right). Though the distance between the amino acids in this case would be only in measure of about three nucleotides, the amino acids could not reach each other yet, especially considering that an amino acid is even much smaller than a single nucleotide.

Now, let us envision the actual structure of a "direct RNA template" (DRT) considering the principle of replication parsimony. First, an aa-binding site in a DRT should have a short sequence, just like a simple aa-aptamer, containing a hairpin loop, a following little stem, an internal (or bulge) loop and another following little stem [18-21]. Next, aa-binding sites in a DRT should be organized in a compact way. However, a one by one setting of them would disfavor amino acids' approaching to each other (Figure 1a). The stereochemical limitation would not come from the encounter of the hairpin loops or internal (or bulge) loops to each other because they could overlap in the three-dimensional space, but come from joints between aa-binding sites (see the empty arrows in Figure 1a), which risks an over-distortion of the RNA's backbone. That is to say, spacer sequences should be introduced at these joints to render the RNA folding (for the amino acids' approaching) possible (Figure 1b). The spacer sequences might have included some substructures to aid the folding, however, this would bring along additional replication cost. Therefore, a more possible scene for the compact form was that the spacer sequences was short and nonstructural, and the folding was directly aided by the interaction between the aa-aptamer domains, e.g. some base pairs between the hairpin loops in the aptamer domains.

How DRTs could appear is a side but important issue for an intact description of the scenario. I have discussed it in Appendix 1, in which it is suggested that DRTs in the compact form mentioned above were not only favored in principle of replication parsimony, but also very likely to have just been the DRTs emerging in the very beginning. In the following, when I mention "DRT(s)", I would mean "compact DRT(s)" of this kind (but note: this does not mean that DRTs must be in this compact form to render the following scenes possible).

In the scene of the DRT mechanism, any specific peptide would cost a large source for the replication of the corresponding DRT (even if in the compact form) --- an aa-binding site would consist of quite a number of nucleotide residues (~15 nt, referring to the structure of a simplest aa-aptamer), and there would be additional spacer sequences to allow the folding. Accompanying the increase of the diversity of peptides for various functions, as well as the increase of the length of peptides for more efficient functions, the replication burden would become greater and greater. Hence, it would be inevitable for the RNA organisms to "look for" a solution to achieve "replication parsimony", under natural selection.

### Emergence of adaptors: the origin of proto-tRNAs and proto-mRNAs

As exemplified by contemporary organisms, a good solution to achieve the replication parsimony is the introduction of adaptors (i.e. tRNA like molecules). In this way, the spacer sequences between the aa-binding sites on a DRT would no longer be necessary --- the stereochemical limitation mentioned above, would be compensated by the spatial stretch of the adaptors (Figure 1c, Left). In addition, for an amino acid, the corresponding "coding" group of nucleotides on the RNA template could be, apparently, much smaller. The formation of peptides would no longer rely on reactions between aa-NMPs (see Appendix 1), but on peptidyl transference between aa-adaptors. The adaptors themselves would bring along some replication cost, but being cost-efficient because they could be used modularly and repeatedly on different DRTs for various peptides.

However, the initial appearance of the adaptors is a problem, considering the myopia of evolution. Apparently, a single adaptor molecule would not work. Two or more adaptor molecules for one kind of amino acids seem also to be of no use, unless homogenous peptides would have some advantages. That is, adaptor molecules should be introduced as a set, being multiple both in number and species. Their emergence could not be attributed to only one or several mutation events. Therefore, there should be an explanation on their original source before they were recruited into the proto-translation system. Interestingly, it was supposed that a tRNA-like structure at 3' end of some single stranded RNA viruses is a relic of the RNA world, wherein it might have served as a "genomic tag" [28,29]. Such tag RNAs were ligated onto functional RNAs to form genomic RNAs and released from genomic RNAs to form functional RNAs repeatedly, and thus should have been abundant in the RNA world and supplied a molecular base for primitive tRNAs' appearance. However, in this "Genomic Tag (GT)" hypothesis, the answer was blurry on how the specificity between amino acids and cognate primitive tRNAs could have been established, which should be a crucial aspect for any feasible mechanism concerning the introduction of adaptors.

In another hypothesis, the "Coding Coenzyme Handles (CCH)", it was suggested that the adaptors might appear primordially as handles for ribozymes to utilize specific amino acids as coenzymes [15,30]. A handle, composed of a nucleotide triplet, could attach onto such a ribozyme by base-pairing. The ligation of the handle with its cognate amino acid would be specified by their affinity. The triplet would turn out as an anticodon. The attraction of this hypothesis is that it offered a simple answer to two complex problems: the origin of adaptors and the origin of genetic code. However, in this idea, the specificity depends heavily on the assumed affinity of amino acids with only three nucleotides (the anticodon triplet), which has never been directly evidenced by experimental work over several decades. Though RNA aa-aptamer experiments have implied such an affinity [20,22,23], obviously, more nucleotides should be involved to ensure the specificity. Additionally, perhaps more important, these simple handles do not seem to be good adaptors. Namely, no spatial stretch was introduced, and amino acids charged on the handles could not reach each other to form peptide bonds yet (Figure, 1c, Right).

**Figure 2:**
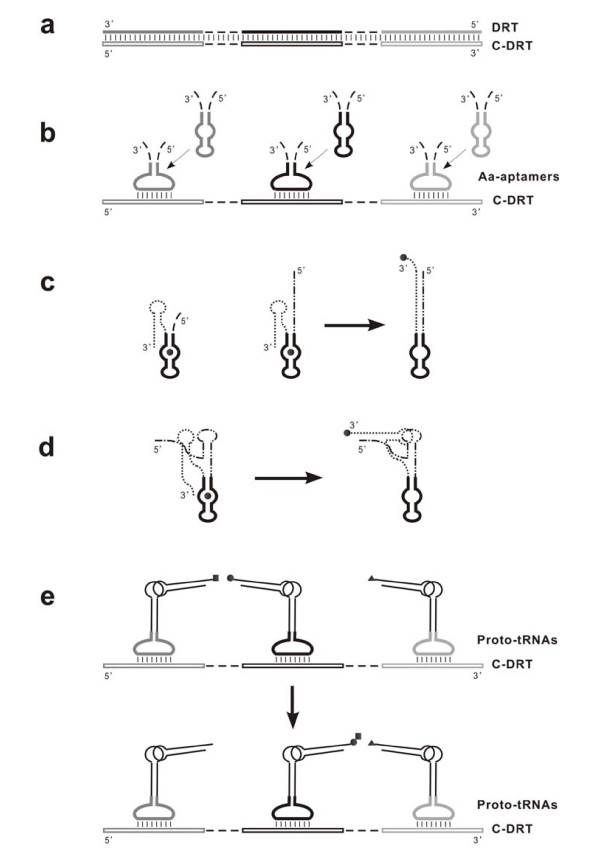
**The emergence of adaptors**. (**a**) The replication of a DRT would produce its complementary strand, "C-DRT". The drawing style of the DRT is the same as that in Figure 1b, except that the aa-binding sites and spacer sequences are drawn in a linear way. In the C-DRT, the complements of the aa-binding sites are correspondingly drawn in dark gray, black and light gray, but as "empty lines", while the complements of the spacer sequences are still drawn as dashed lines. (**b**) Degradation or partial replication of DRTs might result in segments with a core aa-aptamer domain flanked by some spacer sequences. If the aa-aptamer domain could transform into a conformation with a large "recognition loop", the segments might become adaptors using the C-DRT as a template. (**c**) To become a qualified adaptor, the segment's 3'-arm (the dotted line) should fold towards its aa-binding site to accept the amino acid (Left), and then be "grasped" back by its 5'-arm, a simple situation for which could be that the 5'-arm (the dashed-dotted line) is complementary to the 3'-arm (Middle and Right). It should be noted that the adaptors derived from the aa-aptamers in such a way would act on the C-DRT (as shown in **b**) to direct the synthesis of the same peptide synthesized by the DRT. (**d**) The L-shape tRNA-like adaptor --- proto-tRNA. Its 3'-arm (the dotted line), containing the "T-loop", would fold towards its aa-binding site (Left), while its 5'-arm (the dashed-dotted line), containing the "D-loop", could grasp the 3'-arm back via spatial folding (Right). (**e**) The L-shape proto-tRNAs, with a "leg" vertically binding on the C-DRT and an "arm" horizontally delivering amino acids, might turn out to be very suitable for the successive peptide synthesis. Note: the proto-tRNA in **d **is drawn in reference to the real structure of a tRNA, while the proto-tRNAs in **e **are drawn in a simplified form.

Now let us envision the scenario along the line concerning the idea of DRTs. In the DRT hypothesis, it was speculated abstractly that adaptors might have arisen from some aa-binding sequences "escaping" from the RNA templates [20,22,31]. I agree with this speculation, but would supply a detailed interpretation. Due to the instability of RNA, some DRT molecules might degrade; meantime, due to the low efficiency of RNA replicase ribozymes, some replication of DRTs might be aborted before completion. These events might result in segments containing a core aa-aptamer domain flanked by some spacer sequences. On the other hand, complete replication of DRTs would produce corresponding complementary strands (called "C-DRTs" here) (Figure 2a). Apparently, the DRT segments might "recognize" the C-DRTs by base-pairing. Supposing that the aptamer domain of the segments could transform into a conformation with a large "recognition loop" (Figure 2b), they might develop into adaptors using the C-DRTs as templates. The prerequisite is that by some mechanism, amino acids bound by them could be charged onto their chain ends. When the amino acids charged on the chain ends could be brought adjacent spatially, the reaction of peptidyl transference might occur easily [32]. Because the amino acids charged on the end of these adaptors were just those binding on the corresponding sites of the DRT, the C-DRT could direct the synthesis of the same functional peptide synthesized on the DRT. Therefore, less replication turns of the DRT were needed to "supply" the function peptide (the C-DRT is a "necessary part" of the DRT's replication), and the replication cost would be saved. This is a direct advantage, which should have been "within the sight" of the "myopic" evolution (see Appendix 2 for a more detailed interpretation).

Charging an amino acid onto an RNA's chain end seems to be an easy thing, if only the amino acid could be brought adjacent to the chain end, a reaction of which is well within the capacity of ribozymes [33-37]. Wherein, "self-aminoacylation" is a "myopic" mechanism (that could avoid the necessary of the emerge of ribozymes acting *in trans*), provided that the RNA contains an aa-binding site and by some folding one of its chain end could be "located" near the aa-binding site. Quite a few types of such RNAs, with a size only a little longer than typical tRNAs, were derived independently in *in vitro *selection experiments [33]. The rate and aa-selectivity of the reaction could be even greater than those catalyzed by proteinacious AARSs [34]. For a DRT segment mentioned above to charge its binding amino acid onto its 3'-end by this mechanism, its 3'-arm should fold towards its aa-binding site (Figure 2c, Left). But for it to become a "legal" adaptor with sufficient spatial stretch, its 5'-arm should be capable of "grasping" its 3'-arm back to take the charged amino acid away. A simple form could be that the 5' arm is complementary to the 3'-arm (Figure 2c, Middle and Right). Apparently, there might be many alternative forms, in which spatial folding is involved, e.g. the L-shape tRNA like structure (Figure 2d). Considering the diversity or variability of those spacer sequences in DRTs, from which the 3'- and 5'-arms of these segments derived, it could be expected that some of the segments would develop into the qualified adaptors. The L-shape tRNA-like structure, i.e. proto-tRNA, with a "leg" vertically binding on the C-DRT and an "arm" horizontally delivering amino acids, might turn out to be very suitable for the successive peptide synthesis (Figure 2e), thus, over evolution, figuring the final shape of the adaptors.

**Figure 3:**
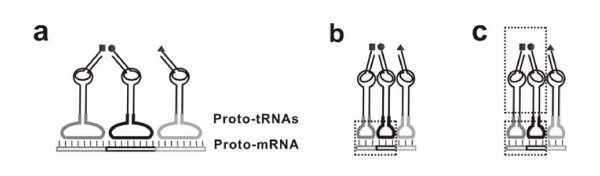
**The emergence of proto-mRNAs and proto-rRNAs**. (**a**) A C-DRT would lose those subsequences between its proto-tRNA binding sites (see Figure 2e) and transform into a "proto-mRNA". (**b**) The emergence of the proto-ss-rRNA (the small dotted-line box) would aid the binding between the proto-tRNAs and the proto-mRNA, allowing the reduction of the base pairs between them, with an ultimate outcome likely to be the triplet anticodon/codon pairs. The large recognition loop of proto-tRNAs would degenerate into a small "anticodon loop". (**c**) The emergence of proto-ls-rRNA (the large dotted-line box) would restrict the amino acids adjacent in a more rigid way, thus favoring the formation of peptide bonds. Note: the "aa-delivering arms" of the proto-tRNAs are drawn with an angle backwards from the present plane.

On a C-DRT, subsequences between "proto-tRNA binding sites" (Figure 2e) would be of no use and tend to lose during evolution in the principle of "replication parsimony". Then, because of their less replication cost, the shortened C-DRTs would gradually take the role of DRTs as the major RNA templates for peptide synthesis, called "proto-mRNAs" here (Figure 3a). Noticeably, the scene described here is a little different from that being anticipated above (see the beginning of this section) --- instead of DRTs themselves, it would be their complementary strands that could bind adaptors and finally develop into proto-mRNAs.

### Towards RNA-based translation: the origin of proto-rRNAs

So far, a proto-tRNA would still recognize a proto-mRNA by quite a few nucleotide residues (Figure 3a). At this stage, this interaction would still be important to ensure the tight binding of proto-tRNAs onto proto-mRNAs. For example, it was experimentally evidenced [38] that if the base pairs between an oligonucleotide and the anticodon loop of a tRNA were reduced from four to three, the association constant would decrease "sharply" from 10^4 ^M^-1 ^to 10^3 ^M^-1^. Indeed, as it was suggested [1](see also [39]), before the emergence of a primitive ribosome, the base pairs between primitive tRNAs and primitive mRNAs should have been more than three to ensure their tight binding. Conversely, it may be envisioned that if an RNA functioning like the small subunit rRNA (called "proto-ss-rRNA" here) could emerge, aiding the binding, the reduction of the base pairs would be allowed. Then, a higher level of replication parsimony would be achieved. The utilization of triplet nucleotide groups seems to be the most parsimonious way, considering the stereochemical relation between the recognition loops of the proto-tRNAs and the proto-mRNA (Figure 3b). Similar to that of proto-tRNAs, the introduction of the proto-ss-rRNA would also be "cost-efficient" because it could be used modularly and repeatedly.

In an experimental research concerning RNA-catalyzed aminoacylation of RNA, it was reported that a specific aa-binding RNA domain contains a cognate codon-anticodon pair [35]. Statistical analysis on the sequence of RNA aa-aptamers suggested that triplet codons or anticodons are "unexpectedly frequent" to appear within cognate aa-binding sites (at least for some amino acids) [20,22,23,31,40], with the case of anticodons turning out to be more evidenced [22,23,31]. Recently, a similar statistical research based on RNA-protein interactions in the ribosome supported that it is anticodons, but not codons, that could interact with cognate amino acids [41]. In fact, the scenario described in the present paper is consistent with the case of anticodons (not that of codons). Namely, only if a proto-tRNA contained anticodon(s) in its aa-binding site, the ultimate triplet remained in the base-pair-reduction mentioned above could be an anticodon (not a codon) --- then, the large recognition loop of a proto-tRNA would degenerate into a small "anticodon loop" (Figure 3b), like that of modern tRNAs.

Certainly, considering the diversity of aa-binding sites for the same amino acids [22,31] and random events possibly involved in the base-pair-reduction, the ultimate remains might also be triplets other than classic anticodons. Then, how could they turn out to be the anticodons finally? First, it may be expected that a uniform genetic code would be fixed within a "lineage" of protocells over evolution because any diversity of the genetic code would seriously interfere with the peptide synthesis. If classic anticodons appear frequently in the aa-binding sites, they would be very likely to turn out as the ultimate remains in the base-pair-reduction, thus figuring the genetic code fixed in most lineages (the majority). Next, the genetic code in all the lineages would finally become universal as the classic one under the mechanism of "innovation-sharing protocols" [42] --- the lineages using the same genetic code with that of majority would benefit most from other lineages' innovations, i.e. new "RNA genes" (transferable between protocells [43]) for synthesizing new functional peptides.

The mechanism of the base-pair-reduction is a side issue, but it is important for the scene described here to be tenable. Here it should be noted that, without a proteinacious AARS, an intact aa-binding site would still be important for a proto-tRNA to charge its cognate amino acid, while some residues in the site would no longer be necessary in the subsequent template-recognition, due to the degeneration of the recognition loop. Then how could this seemingly contradictory situation be settled? I have discussed it in Appendix 3.

The emergence of an RNA functioning like the large subunit rRNA(s), namely "proto-ls-rRNA(s)", would be easier to understand. Supposed that two aa-proto-tRNAs were compactly aligned on a proto-mRNA with the aid of the proto-ss-rRNA (Figure 3b), the peptidyl-transferring reaction might occur but not guaranteed, considering that the fixed point is at the other end (the anticodon loop) and the aa-delivering arms would still have some spatial freedom. The proto-ls-rRNA(s) would restrict the aa-delivering arms in a rigid way to keep the charged amino acids spatially adjacent (Figure 3c), thus working as a ribozyme in a way like that of the large subunit rRNA(s) in modern ribosome [10,11]. In fact, the proto-ls-rRNA(s) might also have emerged before the proto-ss-rRNA, because by restricting the aa-delivering arms, their emergence could already promote the synthesis of a peptide on a proto-mRNA (Figure 3a), or even earlier, on a C-DRT (Figure 2e).

It should be noted that though the modern small subunit rRNA (e.g. 16 S rRNA) and large subunit rRNAs (e.g. 23 S rRNA) are complex molecules composed of nucleotide residues over one or more thousand, biochemical and genetic researches have suggested that their primitive form in the RNA world might have been much simpler, composed of nucleotide residues only around one hundred or even less [44]. A recent structural analysis on 23 S rRNA confirmed that its evolution should have "started with an initial fragment of about 110 nucleotides" [45]. This size is just that of a small or ordinary ribozyme. Therefore, the proto-ss-rRNA and proto-ls-rRNA(s) could have derived from other functional RNAs (ribozymes) via mutation, without large obstacles. In other words, their emergence should have been well within the sight of evolution.

Thereafter, a mechanism of the proto-rRNAs' translocation on a proto-mRNA (composed of triplet codons then) should have emerged, with the advantage of avoiding repeated disassembly and assembly of the proto-rRNAs when the proto-translation reaction turned to subsequent codons.

Accompanying the emergence of the whole proto-translation system, with much shorter template RNAs and greater efficiency than that in the case of the DRT mechanism, more and longer useful peptides would emerge under natural selection. Finally real proteins with great structural and chemical diversity would emerge, many of which would act as independent functional molecules, taking the role of most functional RNAs. Wherein, certainly, the proto-translation system would incorporate proteinacious components gradually, and evolve towards the modern translation system. The primary life was thus leaving from the RNA world and arrived in the RNP world.

## Testing the hypothesis

**Figure 4:**
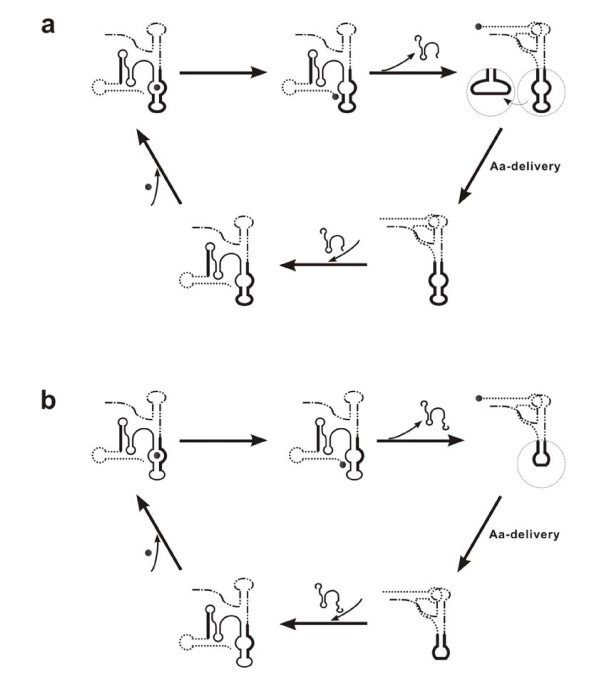
**The degeneration of the proto-tRNAs' recognition loop might be through the extension of an inserted self-splicing intron**. (**a**) The self-splicing intron might have been inserted as an *in cis *"aminoacyl-tRNA synthetase" ribozyme for proto-tRNAs. By self-splicing and self-inserting, the intron could mediate the interchange of the self-aminoacylation state and the charged mature state of a proto-tRNA. The drawing style is the same as that in Figure 2d, except that the thin solid line, denoting the intron, is added. The aa-binding site of a proto-tRNA (circled in the top-right subfigure) could transform into a large recognition loop to recognize a template RNA. (**b**) With the extension of the self-splicing intron, the residues no longer necessary in the template-recognition would be removed accompanying the intron's splicing. The proto-tRNA would then recognize a template RNA by a small anticodon loop (circled in the top-right subfigure). The inserting point of the intron was adopted in reference to experimental evidence [77-79]. Note: the intron was not drawn in the same scale as the proto-tRNA, and was also not drawn according to the real structure of a self-splicing intron, which might be much more complex.

The scenario described here is open for experimental work at several key scenes. First, it is possible to demonstrate a (compact) DRT mechanism (Figure 1b). We might synthesize an RNA containing multiple aa-binding sites (for active amino acids, e.g. aa-AMPs) and capable of bringing the amino acids adjacent via folding (with the involvement of spacer sequences), and then detect if a corresponding peptide could be synthesized with this RNA in a solution of such active amino acids. Second, it might not be difficult to show if adaptors, capable of self-aminoacylation, could be constructed from aa-aptamers (Figure 2c), based on the work concerning the self-aminoacylation of simple RNA molecules [33]. Third, the possibility of synthesizing a peptide by proto-tRNAs and a proto-mRNA, without the participation of proto-rRNAs (Figure 3a), might be demonstrated --- it may be possible to modify a tRNA by introducing an aa-binding sequence into its anticodon loop to mimic a proto-tRNA, while to synthesize a "proto-mRNA" containing corresponding complementary subsequences for such "proto-tRNAs" to recognize. Fourth, the model concerning self-splicing intron's role in the inter-transformation of the self-aminoacylation state and the mature state of proto-tRNAs (Figure 4a), as well as its "modified version" after the base-pair-reduction between proto-tRNAs and proto-mRNAs (Figure 4b), might also be tested by experimental work, under appropriate designs.

It may be not easy to demonstrate experimentally the evolving processes driven by replication parsimony in the scenario, which should have lasted a rather long period in early stages of life. However, it could be expected that computer simulation would help us to evaluate the plausibility of these processes. As a parallel example, our previous computer simulation studies concerning the origin of the RNA world demonstrated that the limitation of replication resources should have been an important selective pressure for the evolution in the RNA world [46-48]. More interestingly, it was shown recently by computer simulation that the triplet genetic code could have evolved from larger size codes, with the advantage of saving "resources and time" in replication [39]. This evolving process, driven by "replication parsimony", just corresponds to one of the scenes in the scenario described here --- the base-pair-reduction between a proto-tRNA and a proto-mRNA, accompanying the emergence of proto-ss-rRNA that could stabilize their inter-binding (Figure 3, from a to b). In future, perhaps with the instruction of *in silico *results like this, some experiments (e.g. based on *in vitro *molecular evolution) could be designed to test these evolving processes involving "replication parsimony", at various aspects.

## Implication of the hypothesis

### A comparison with other hypotheses on the origin of translation

As mentioned in the "Background", there have been a number of hypotheses on the scenario about the origin of translation (protein synthesis) in the RNA world. Here I will compare the present one with fourteen of them, which are deemed representative (Table 1).

**Table 1:** A comparison of hypotheses on the scenario of the origin of translation.

Hp: No. name [ref.] Author(s)	Peptides' specificity	tRNAs		mRNAs		rRNAs		aa-tRNA specificity
			
		**Ad**.	**Or**.	**Ad**.	**Or**.	**Ad**.	**Or**.	
1. DRT [20,22] Yarus et al.	+^▲^	+	O**^▲^**	-	O^▲^	O	O	O^▲^

2. GT [28,29] Weiner & Maizels	O	O	+	-	-	-	-	-

3. CCH [15,30] Szathmary	O	O	+	+	-	-	-	+^▲^

4. TR [53] Gordon; [54] Poole et al.	O	+	+	-	O	O	+	-

5. [16] Wolf & Koonin	+	+	+^▲^	-	O	+	+	O

6. [1] Crick et al.	O	O	-	-	-	O	-	-

7. [55] Knight & Landweber	O	O	O	O	-	-	-	+

8. [56] Di Giulio	O	O	O	-	-	O	-	-

9. [57] Noller	O	O	-	-	-	O	+^▲^	-

10. [50] Brosius	-	O	+	-	O	O	+	-

11. [51] Schimmel & Henderson	-	O	+	-	O	O	-	-

12. [52] Bernhardt & Tate	-	O	+	+	O	O	-	-

13. [39] Baranov et al.	-	O	+	-	O	O	-	-

14. [49] Yakhnin	N/A	+	+	-	O	O	+	-

▲ DRT-RP	+	+	+	+	+	+	-	+

The names of hypotheses: DRT (Direct RNA Templating); GT (Genomic Tag); CCH (Coding Coenzyme Handles); TR (Triplet Replicase); ▲DRT-RP (Direct RNA Template - Replication Parsimony), the present hypothesis; the others were not given a name. For tRNAs, mRNAs and rRNAs, "Ad." means "the direct advantage of their emergence", while "Or." means "the original source of their emergence". In general, "+" means "clearly stated"; "-" means "not stated"; "O" means "implied or stated in an abstract way"; "N/A" means "Not Applicable"; "^▲" ^means "associated with the present hypothesis". See text for detailed explanations.

Usually, the hypotheses were quite different on various aspects of the scenario. For example, even the views on the relationship between primordial peptides and the emergence of proto-translation system were not consistent. A recent hypothesis asserted that the driving force for the emergence of a proto-translation system is associated with the double strand-dissociation in the RNA replication process, having nothing to do with primordial peptides (the 14^th ^hypothesis in Table 1, i.e. Hp14)[49]. Certainly, most of the hypotheses admitted that primordial peptides should have some advantages for protocells and thus figure the "ultimate driving force" for the emergence of a proto-translation system. However, a portion of them asserted or assumed that primordial peptides should have no (or little) sequence specificity (Hp10-13)[39,50-52], namely, non-specific (homogenous or random) peptides might have supplied the advantages in the beginning. Others asserted or implied that the primordial peptides should have sequence-specificity to be functional (Hp1-9)[1,15,16,20,22,28-30,53-57]. Following the DRT hypothesis (Hp1), the present one, which could be named the "DRT-RP" ("RP" for "replication parsimony") hypothesis, asserts that the primordial peptides should have sequence-specificity, thus avoiding a speculation upon the function of non-specific peptides (see [52] for a speculation on the possible function of polyglycine, and also [58] for a speculation for the possible function of nonspecific peptides enriched with positively charged amino acids).

Considering the myopia of evolution, the major components of a proto-translation system, proto-tRNAs, proto-mRNAs and proto-rRNAs, should not have emerged simultaneously. In other words, they should have been introduced into the system in intermediate steps. Therefore, the direct advantage for the introduction of each of them should be explained. In addition, if a component should be introduced in a "complex form", which could not have occurred via some "ordinary mutation events", there should be an explanation on its original source. The original source should have been some RNAs bearing other functions previously. This evolving way could be termed "exaptation" [16].

The introduction of adaptors is the most striking event in the origin of the translation system. Usually, the advantage of the introduction was just implied as "producing primordial peptides" (Hp2, 3, 6-13). In other words, without adaptors, no peptides could be synthesized. In Hp1 (DRT) and Hp5, it was asserted that initially, peptides could be synthesized without adaptors. In the former, the advantage of the introduction of adaptors was to "adapt amino acids to" their binding sites on DRTs, while in the latter, to improve "the stability and spatial precision" of the binding of amino acids on the ribozyme facilitating their ligation. According to Hp4 and Hp14, proto-tRNAs was first involved in the RNA replication, in which the advantage of their emergence was implemented. In agreement with Hp1 and Hp5, the present hypothesis (DRT-RP) asserts that primordial peptides were synthesized without adaptors. However, it states that the advantage of the introduction of the adaptors should be to exploit C-DRTs to synthesize the peptides originally synthesized only by their corresponding DRTs, thus saving the replication cost for the DRTs (because a C-DRT is a necessary part in the replication of its corresponding DRT).

As mentioned above, the adaptors should be introduced "as a set", being multiple both in number and species. This is just a "complex form", the appearance of which could not be attributed to only one or several mutation events. Therefore, there should be an explanation on their original source before they were recruited into the proto-translation system. The most popular explanation came from the "Genomic Tag (GT)" hypothesis (Hp2) (see above in the section "*Emergence of adaptors: the origin of proto-tRNAs and proto-mRNAs*" for the explanation), which has been adopted in several other hypotheses (Hp10, 11, 14). The other two explanations came from the "Coding Coenzyme Handles (CCH)" hypothesis (Hp3) (see above in the section "*Emergence of adaptors: the origin of proto-tRNAs and proto-mRNAs*" for the explanation) and Hp5 (concerning "retention of amino acids in protocells", see Appendix 2). The DRT hypothesis (Hp1) had an abstract explanation, which stated that the adaptors should have "escaped" from DRTs. The present hypothesis (DRT-RP) formulates a detailed explanation for the "escaping" (Figure 2). Meanwhile, it is explained that the original source of the adaptors might also have come from the aa-repository RNAs (see Appendix 2), following the view in Hp5.

The direct advantage of introduction of proto-mRNAs could be to direct the synthesis of specific peptides, if specific adaptors for amino acids had been "invented" before the emergence of peptide synthesis, as asserted in the CCH hypothesis (Hp3) and Hp7. However, if this is not the case, the direct advantage for their introduction might not be associated with this aspect. For example, it was stated in Hp12 that the direct advantage for the initial appearance of proto-mRNAs is to enhance the stability of proto-tRNAs' binding on an "ancestral peptidyl transferase ribozyme" (a primitive form of proto-ls-rRNA). In the present hypothesis (DRT-RP), the advantage of the evolving process from C-DRTs (Figure 2e) to proto-mRNAs (Figure 3a) is attributed to the saving of replication cost for the templates that direct the synthesis of functional peptides.

Unlike proto-tRNAs, proto-mRNAs could be introduced into the translation system "independently". Thus, their original source has not an urgency to be interpreted clearly, especially considering that they might not be long, corresponding to those short primordial peptides. Nevertheless, there have been some abstract explanations to this point (Hp1, 4, 5, 10-14). Wherein, it was usually implied (more or less) that proto-mRNAs might have arisen from RNAs with a random sequence. The DRT hypothesis (Hp1) is an exception, which suggested that proto-mRNAs might have come from the specific RNAs, DRTs, which could "direct" the synthesis of primordial functional peptides. The present hypothesis (DRT-RP) agrees to this viewpoint largely, but explains in a clear way that proto-mRNAs should have actually come from complementary chains of DRTs (i.e. C-DRTs).

Due to solid evidence on the role of rRNAs in the modern translation systems, there seems to be little controversies on the advantage of the introduction of proto-rRNAs (Hp1, 4-6, 8-14). Wherein, the proto-ss-rRNA would stabilize the association of proto-tRNAs with a proto-mRNA, and the proto-ls-rRNA(s) would facilitate the peptidyl transference between proto-tRNAs. The present hypothesis (DRT-RP) agrees with this viewpoint, but emphasizes that with the stabilization function, the proto-ss-rRNA would allow the reduction of base pairs between proto-tRNAs and proto-mRNAs. Thus, proto-mRNAs could become shorter (from Figure 3a to 3b), and their replication cost could be saved to some extent.

The original source of rRNAs has attracted much attention (Hp1, 4, 5, 9, 10, 14), often occupying an important role in the hypotheses. The hypothesis of "Triplet Replicase (TR)" (or "Triplicase", Hp4) stated that proto-rRNAs should have come from an RNA replicase, which cut the anticodon triplets from proto-tRNAs to be used as substrates in the template-directed RNA replication. Similarly in some degree, Hp14 also asserted that proto-rRNAs should have had its origin in the RNA replication. It stated that the proto-ls-rRNA(s) should have derived from an RNA polymerase replicase (using monomers as substrates), while the proto-ss-rRNA should have derived from a partner of this replicase, which could prevent the annealing of the copy and template strands (thus facilitating a new turn of replication). Hp5 asserted that the proto-ls-rRNA(s) should have derived from a ribozyme using two or more amino acids as coenzymes, but did not offer a clear explanation of the original source of the proto-ss-rRNA. Hp9 speculated that proto-rRNAs might have derived from group I intron. Hp10 stated that proto-rRNAs might have derived from an ancient form of a bacterial RNA, named "tmRNA", which contains separate domains that can serve as a tRNA and an mRNA. The DRT hypothesis (Hp1) asserted that proto-rRNAs might have derived from intermediate subsequences between aa-binding sites on a DRT, because these intermediate subsequences had a structure, and thus a function, to facilitate the ligation of amino acids on the aa-binding sites. However, it seems hard to imagine how these intermediate subsequences could still function in such a way when they "escaped" from the DRT, as independent RNA molecules. Moreover, it seems that DRTs would be most likely to have emerged in a compact form (Appendix 1), in which these intermediate subsequences should be just short "spacer sequences", perhaps without substructures (see Figure 1b). Therefore, the present hypothesis (DRT-RP) tends to disagree with the opinion of the DRT hypothesis at this point. In fact, unlike proto-tRNAs, which should be introduced "as a set" (being multiple both in number and species), the proto-rRNAs would "stand alone", especially considering that the proto-ss-rRNA and proto-ls-rRNA(s) might have been introduced separately, due to different advantages. Hence, their introduction would not be in a "complex form" unless their molecular size (or say, structure) would be extraordinarily large. However, there was evidence showing that both the proto-ls-rRNA(s) and proto-ss-rRNA might have been small RNAs, with a sequence length around one hundred nucleotides or even less [44,45] (see the section "*Towards RNA-based translation: the origin of proto-rRNAs*"). In other words, they could have been derived from some ordinary ribozymes acting in the RNA world, via some "ordinary mutation events" Therefore, an explanation for their original source seems to be not so urgently needed as it has often been deemed. On this consideration, such an explanation is not clearly offered in the present hypothesis. Certainly, if there should indeed be a speculation on the original source of the proto-rRNAs, I tend to agree the suggestion in Hp9, namely that proto-rRNAs (especially proto-ls-rRNAs) might have derived from group I intron (for the reason, see the last paragraph in Appendix 3).

Of all the previous hypotheses, Hp5 is the most impressive on its efforts to describe the whole scenario in detailed intermediate steps, considering the "myopia of evolution". Namely, almost all the advantages and original source of proto-tRNAs, proto-mRNAs and proto-rRNAs were considered within (see Table 1). Another impressive one at this aspect is Hp14, though it asserted that all these components might have been recruited from the RNA replication. The present hypothesis (DRT-RP), is also formulated in such a way, by which I believe would be beneficial both for a comprehensive understanding on its logic and for a detailed design of experiments to test it (see the part "Testing the hypothesis"). Another point that should be noted here is that though the present hypothesis viewpoints are similar or overlapped with some previous hypotheses on the original sources of proto-tRNAs, proto-mRNAs and proto-rRNAs (see the superscript "^▲^" in Table 1), its viewpoints on the direct advantages of their emergence is unique. Most importantly, all these advantages (except that of proto-ls-rRNAs) are believed to be associated with one reason, namely, to save the replication cost for the RNA templates directing the synthesis of functional peptides. This "replication parsimony" principle might have figured the major clue within the whole scenario on the origin of translation. By contrast, no previous hypotheses have ever described such a consistent clue, or something alike.

Finally, an indispensable issue in the origin of the proto-translation system is how the specificity between amino acids and their cognate adaptors could have been established, which is associated with the origin of the genetic code (see below). The problem is a critical one for the hypotheses assuming that the specificity of primordial peptides is important (Hp1-9), and at least a necessary one that should be solved sooner or later in the scenario described in others (Hp10-14). However, only a few of them addressed the problem directly (Hp1, 3, 5, 7). The CCH hypothesis (Hp3) attributed the specificity to the chemical affinity between amino acids and their cognate anticodon (see the section "*Emergence of adaptors: the origin of proto-tRNAs and proto-mRNAs*"). The main problem comes from that there is not evidence shown the affinity to be strong enough to ensure the specificity, and that the coenzyme handles do not seems to be good adaptors with sufficient spatial stretch to allow their charged amino acids to reach each other (Figure 1c, right). The DRT hypotheses (Hp1) and Hp7 attributed the specificity to the chemical affinity between amino acids and their RNA binding sites enriched with cognate anticodons/codons, in which Hp1 did not make a clear choice upon anticodons or codons, while Hp7 chose codons unambiguously. Hp5 admitted the possibility of such a scene but did not insist that it ought to have occurred in the scenario of the origin of translation. Following the DRT hypothesis, the present hypothesis (DRT-RP) agrees to this scene, but chooses anticodons unambiguously (at this point, like the CCH hypothesis).

### The compatibility with theories on the origin of the genetic code

The origin of the genetic code is a problem tightly associated with the origin of proto-translation system. Because the latter is "notoriously difficult", quite a number of hypotheses concerning the origin of the genetic code were based on formal schemes (for reviews please see [59,60]). The hypotheses could be generally classed into four categories: first, the stereochemical theory (e.g. [4,7,15,20,22,23,30,40,41,55,61]), which stated that interactions between amino acids and their cognate anticodons/codons are the most important factor that shaped the genetic code; second, the coevolution theory [62-64], which stated that the genetic code formed initially for simple, prebiotically available amino acids and then gradually for complex, biosynthetic amino acids; third, the adaptive theory (e.g. [65-72]), which stated that the genetic code evolved in a way to minimize the possible confusion or errors in translation; fourth, the frozen accident theory [6], which stated that the extant genetic code came from the fixation of an early (perhaps random) code because any change in the code would result in significant changes in all functional proteins and, consequently, would be lethal. Apparently, the four theories tend to tackle different aspects of the formation of the genetic code and, thus, might be compatible [59,60].

As mentioned above, the issue of "formation of aa-tRNA specificity" (Table 1) is just the point at which the origin of translation is associated with the origin of the genetic code. In fact, the origin of the genetic code can be rephrased as "the formation of primary aa-tRNA specificity and its subsequent evolution to the extant aa-tRNA specificity in contemporary organisms". It seems likely that the stereochemical theory might have shaped the "formation of primary aa-tRNA specificity", the coevolution theory and the adaptive theory might have governed "its subsequent evolution", and the frozen accident theory might had have some associate role in both the two stages. Actually, the hypotheses on the origin of translation (in Table 1) that tried to explain the formation of primary aa-tRNA specificity (Hp1, 3, 5, 7) all approved the stereochemical theory, except that Hp5 also included an explanation based on the frozen accident theory. The other hypotheses implied in them more or less a view of the frozen accident theory. Meanwhile, all the hypotheses did not exclude the possible roles of "coevolution" and "adaptation" in the subsequent evolution of the aa-tRNA specificity. Clearly, the present hypothesis (DRT-RP) approves the stereochemical theory, but does not exclude the possibility of a subsequent optimization or frozen accident.

However, some surprisingly, one could find that the present (DRT-RP) hypothesis tend to adopt a different view from the coevolution theory. That is, the initial amino acids that entered the genetic code should be complex, biosynthetic amino acids instead of simple, prebiotic amino acids. In this hypothesis, it is believed that the retention of amino acids in protocells for their repetitive usage was important and could be achieved by RNA-binding. Such RNA-binding of amino acids would result in the origin of DRTs (see Appendix 1) and perhaps also that of adaptors (see Appendix 2). Apparently, complex amino acids, which were "biosynthesized" within the protocell, would be more precious than simple amino acids, which were available in the prebiotic circumstance. Additionally, complex amino acids would be more important considering their possible function, e.g. as coenzymes of ribozymes. Along this logical line, the first aa-repository RNAs, including RNAs with multiple aa-binding sites (subsequently evolved into DRTs) and single aa-aptamers (subsequently developed into adaptors), should have just emerged for the retention of those complex amino acids. As a consequence, the first peptides should have been composed of complex amino acids, and the anticodons/codons for the complex amino acids should have emerged first in the genetic code. Later, accompanying the exhaustion of simple amino acids in the prebiotic circumstance, they also had to be "biosynthesized" within protocells and the retention of them would become important. Then aa-aptamers for these simple amino acids would emerge and, subsequently, develop into corresponding specific adaptors Thereafter, these simple amino acids would find their place in the peptides, perhaps acting as structural elements to allow the emergence of more complex functional peptides and, finally, proteins. Such a scene is consistent with the present evidence that only the aa-aptamers for complex amino acids, e.g Arg, His, Ile, Phe, Trp and Tyr, have been found to be enriched with cognate anticodons (/codons) [22,23,31]. This list shares nothing with that of popularly admitted simple, prebiotic amino acids, e.g. Gly, Ala, Ser, Asp and Glu. Remarkably, a recent statistical research based on RNA-protein interactions in the ribosome confirmed this situation [41]. It was shown in this research that simple, prebiotic amino acids has no relation to their anticodons (except Asp), but those complex amino acids tend to be related to their anticodons significantly. A reasonable deduction was that complex amino acids should have entered the genetic code through aa-anticodon interactions, but simple amino acids should have not [41]. However, according to the common sense that simple amino acids should have been early arrivers in the genetic code [62-64], the authors "had to" suggest that the genetic code was reassigned after the arrival of complex amino acids [41]. Apparently, the reassignment might cause the "lethality" that was asserted in the frozen accident theory [6], thus no quite plausible. Here, if we agree that the complex amino acids would arrive earlier than the simple ones, this reassignment could then be avoided. Simple amino acids should have been introduced into the genetic code later, under the principle of "not conflicting with those of complex amino acids". Consequently, even if a simple amino acid might have some stereochemical relation with a nucleotide triplet, the triplet might not turn out to be its anticodon. Therefore, we could see the extant genetic code table --- complex amino acids and their cognate anticodons were often observed at the RNA-aa (protein) binding interface, but for simple amino acids, this is not the case.

## Conclusion

The present hypothesis (DRT-RP) provides a major clue for the scenario on the origin of translation in the RNA world --- it should have started with the Direct RNA Template (DRT) mechanism [20,22], and evolved towards an RNA-based proto-translation system in the principle of "replication parsimony". In more detail:

1) The DRT mechanism was the simplest mechanism for RNA to synthesize peptides, and thus very likely to have been adopted initially, but the increase of the diversity and the length of the peptides would be seriously limited because the replication cost of DRTs was great, even if the DRTs could be organized in a most compact way;

2) Adaptors (proto-tRNAs) would arise to allow the exploitation of the complementary strand of a DRT (a necessary part in the DRT's replication, called "C-DRT" here) to synthesize the same functional peptide synthesized by the DRT, thus saving the replication cost (i.e., for the synthesis of definite copies of the peptide, less turns of the DRT's replication would be needed);

3) Acting through the adaptors (with sufficient spatial stretch), these C-DRTs could transform into much shorter templates (called "proto-mRNAs" here), and substitute the role of original template RNAs (DRTs), thus significantly saving the replication cost;

4) The proto-rRNA corresponding to small subunit rRNA (proto-ss-rRNA) would then emerge to aid the binding of the proto-tRNAs on the proto-mRNAs, allowing the reduction of base pairs between them (ultimately resulting in the triplet anticodon/codon pair), thus further saving the replication cost.

In this context, the replication cost saved would allow the appearance of more and longer functional peptides and, finally, proteins. The logic is simple, clear and reasonable. Especially, it is well consistent with the fundamental idea concerning the scientific interpretation of the origin of life --- chemistry in nature does not exist with an aim to support life, instead, life would "manage" to exploit potential chemical resources (in this case, peptides and proteins), eventually in an efficient way over evolution, under the power of natural selection.

## Competing interests

The author declares that they have no competing interests.

## Appendices

### Appendix 1

#### How could DRTs appear?

The answer that initially RNA would bind amino acids just in order to make beneficial peptides would fall back on the assumption that evolution worked like an engineer, thus not appropriate. A feasible answer could be formulated as the following. Amino acids might have acted as coenzymes to offer some missing functional groups for ribozymes [15,30], or acted as substrates in the synthesis of nucleotides or other important molecules in the RNA world [73]. Additionally, some amino acids with positive charge might stabilize RNA [40], which has a negative backbone. Hence, it is reasonable to suggest that RNA might bind amino acids to retain them in protocells for repeated usage [16], considering that they are small molecules tending to diffuse across the membrane. In other words, there might be an RNA repository for amino acids in protocells (like sarcoplasm reticulum for calcium ions in muscle cells).

In the repository RNAs, there might be a portion containing multiple aa-binding sites, for which the benefit was that one turn of RNA replication would produce more aa-binding sites (like the situation of tandem-organized rRNA gene clusters or histone gene clusters in modern genomes). However, if the multiple aa-binding sites were organized one by one as shown in Figure 1a, apparently only a few aa-binding sites would be allowed, also due to the stereochemical limitation concerning over-distortion of the RNA's backbone. Namely, some spacer sequences should be introduced to incorporate more aa-binding sites into a single RNA molecule. Thus, these RNAs with multiple aa-binding sites would be very similar to that of "compact" DRTs (Figure 1b), the emergence of which would then be "within the sight" of evolution.

For amino acids to be ligated to form a peptide, some sort of energy source was required. In an RNA-based protocell, active nucleotides, e.g. NTPs, would be abundant for the synthesis of RNA. The reaction of NTPs with amino acids would form aa-NMPs, which could be catalyzed by ribozymes [74]. There might be several reasons for the formation of these activated amino acids. First, they might be needed in the synthesis of nucleotides or other important molecules [73]. Second, aa-NMPs would be more difficult to diffuse through the membrane, thus also contributing to the retention of amino acids. Third, the NMP group could aid the amino acid's binding onto RNA [34], thus favoring the "job" of repository RNAs mentioned above. Hence, it comes to us a scene that the amino acids binding on RNA might be actually in an activated form, and the formation of peptide linkages would be energetically favored.

Comparing with single amino acids, some peptides might be more efficient to aid the activities of ribozymes [75,76], or to stabilize RNA structures. Additionally, due to the incorporation of both the hydrophobic and hydrophilic amino acids, some peptides might work on the membrane of protocells, aiding the incoming of raw materials. Provided that such roles of peptides were important for RNA-based protocells, some multiple aa-binding (repository) RNAs favoring the formation of these peptides would be selected out and become DRTs, Note that DRTs emerging in this way would just be the compact DRTs, and this scene suggests that the compact DRTs were not only favored in the principle of replication parsimony, but also very likely to have just been the DRTs emerging in the very beginning.

### Appendix 2

#### The emergence of adaptors considering the myopia of evolution

It is assumed here that adaptors would come from the degradation or partial replication of DRTs, however, the adaptors would have to be aminoacylated before they could conduct their function. Therefore, in a view of "evolution has no foresight", an alternative explanation on the original source of the adaptors, already charged with cognate amino acids, might be more convincing.

In the aa-repository RNAs (see Appendix 1), beside the ones containing multiple aa-binding sites, which could have developed into DRTs, there could also be single aa-aptamers. If, as it was assumed, retention of amino acids within protocells was very important [16], such single aa-aptamers might transfer their binding amino acids to their chain ends to form a covalent form (perhaps by self-aminoacylation, see text), for a better retention. Significantly, just as free active amino acids (e.g. aa-AMP), amino acid residues covalently charged on the RNAs' chain end is also energetically activated for reactions that might involve the amino acids [73]. Such a scene has been described in [16] (see also [73]). If this was the case, there would have been an abundant source of the adaptors, already charged at their chain ends with cognate amino acids.

However, it should be noted that these adaptors were not directly derived from DRTs. For their binding onto C-DRTs, it should be assumed that there was only one species of aptamers (thus adaptors) for each kind of amino acids in a protocell, or that different aptamers for the same amino acid would adopt similar sequences. The first assumption is reasonable considering that aptamer molecules for one specific amino acid might have descended from a common ancestor RNA by duplication. The second assumption may also be reasonable considering that sequences of different RNA binding sites for the same amino acid seem enriched with its anticodon(s) [22,23] (see the section "*Towards RNA-based translation: the origin of proto-rRNAs*" for details), and that the binding between the adaptors and a C-DRT (Figure 2b, e) might be able to "tolerate" partial mis-base-pairing to some extent.

### Appendix 3

#### The reduction of base pairs between a proto-tRNA and a proto-mRNA

The base pairs between a proto-tRNA and a proto-mRNA could reduce when the proto-ss-rRNA emerged, aiding their binding to each other. The mechanism of the base-pair-reduction needs an interpretation. Here it should be noted that an intact aa-binding site would still be important for a proto-tRNA to charge its cognate amino acid, while some residues in the site would no longer be necessary in the subsequent template-recognition. Then how could this seemingly contradictory situation be settled? Actually, the structure of the proto-tRNAs' aa-binding site (the aa-aptamer domain) is very similar to that of the intron-contained anticodon loop of some modern tRNA precursors in eukaryotes and archaea [77], particularly in respect of the internal (or bulge) loop that is included. If we consider the intron-contained anticodon loop as a relic from the RNA world, the situation might have been settled by a mechanism of intron-removing. Namely, the residues no longer necessary in the template-recognition would be removed as an intron after the completion of aa-charging. However, if the intron's removal would involve some other ribozymes (acting *in trans*), the mechanism seems not easy to appear considering that evolution "has no foresight". Interestingly, it was revealed that the intron within some tRNA precursors in plastids, mitochondria and bacteria is a larger, self-splicing one (the group I or group II intron) [78-80], which might be more ancient [77,81] and perhaps have had some role in the development of proto-tRNAs [30]. Then, what a mechanism should it be if the case of a self-splicing intron is considered?

Now let us turn back to an earlier stage, before the emergence of the proto-ss-rRNA. The self-aminoacylation state of proto-tRNAs might be not very stable (Figure 2d, Left) and have a tendency to change to the L-shape state (Figure 2d, Right) before completion of the self-aminoacylation. The introduction of a self-splicing intron might be helpful to stabilize the self-aminoacylation state (Figure 4a, Top-left). The self-splicing and self-inserting (reverse splicing) of the intron would mediate the interchange of the self-aminoacylation state and the charged mature state (Figure 4a). Conceptually, the self-splicing intron could be deemed as an *in cis *"aminoacyl-tRNA synthetase" ribozyme for the proto-tRNAs. There has been experimental work suggesting that group I intron can catalyze the aminoacyl transfer reaction [82]. More interestingly, it was revealed that some mitochondrial aminoacyl-tRNA synthetases (AARSs, proteinacious) in fungi may aid the self-splicing of group I introns [83-85]. In the context of the present discussion, an intriguing explanation could be formulated as: group I introns might have existed in proto-tRNAs, and proteins (peptides) aiding their self-splicing emerged later, which finally evolved into AARSs, and substituted the role of the introns in favoring the synthesis of aminoacyl-tRNAs.

If the model shown in Figure 4a could be accepted, it would be natural to accept a following idea. Namely, upon the emergence of the proto-ss-rRNA, when a proto-tRNA could bind onto a proto-mRNA with less base pairs, the residues in its aa-binding site no longer necessary in the template-recognition would be incorporated into the self-splicing intron and removed accompanying the splicing. The extension of the intron should have been an "easy" event, considering that variation of introns, in particular their length, is ordinary. Then the model would have its modified version in this stage (Figure 4b), wherein the contradictory situation mentioned above was settled.

Comparing the structure of the self-aminoacylation state (Figure 4a, Bottom-left, Top-left and Top-middle) and that of the mature state (Figure 4a, Top-right and Bottom right), we could notice that the group I intron (thin solid line) should share a common subsequence with the aa-binding site (thick solid line). Additionally, the common subsequence would extend accompanying the extension of the intron (Figure 4b). Interestingly, it has been demonstrated that the group I intron in the extant ribosome can specifically bind arginine (by the same part of the RNA which binds the guanosine co-factor) [86]. This could be deemed as a support to the model presented here if we assume that the partial arg-binding site could also bind arginine to some extent. Moreover, it implies that the ribosomal group I intron might have derived from the group I intron that served as an *in cis *"arg-tRNA synthetase" ribozyme. Further, such an association might bear some potential supports for the hypothesis stating that even proto-rRNAs (especially the proto-ls-rRNAs) themselves might have evolved from ancient group I introns [57].

## Reviewers' comments

I am grateful to the reviewers for their thoughtful analysis and critique of my manuscript. I think that this manuscript, which addresses a challenging problem full of controversies, is very suitable to appear in this journal, which has a policy to publish reviewers' comments and authors' responses together with a manuscript. In my response to the reviewers below, I have omitted some minor points brought to my attention (language, additional references, formatting, etc.), correcting them directly in the manuscript instead.

**Reviewer 1: **Eugene V. Koonin, National Center for Biotechnology Information, NIH, USA

This manuscript addresses what I believe to be the ultimate problem in the study of the origin of cells: the emergence of the translation system. Any informed discussion of this fundamental enigma is of interest, and this one is no exception. I find it equally evident that the decisive breakthrough remains elusive, and even the direction in which one should proceed to find it is less than obvious. Again, this manuscript is no exception because, in my opinion, it makes very little progress towards a plausible solution.

**Author's response: **Yes, the emergence of the translation system is almost the most challenging problem in the origin of life. According to the RNA world hypothesis, RNA could both self-replicate (via Watson-Crick base-pairing) and act as functional molecules in an early stage of life. The key issue is how a completely different set of molecules, namely proteins, could emerge to substitute the functional role of RNA. Apparently, an RNA-based "proto-translation system", perhaps including proto-tRNAs, proto-mRNAs and proto-rRNAs, would be too complicated to emerge at one step. Though a number of hypotheses have attempted to explain those intermediate steps, they lacked a major clue. Just as the reviewer said, "even the direction in which one should proceed to find it is less than obvious". The present hypothesis is just an effort to illustrate the direction, i.e. if starting from the DRT mechanism, which should be the simplest and most direct mechanism for RNA to synthesize peptides, the proto-tRNAs, proto-mRNAs and proto-rRNAs (in fact, the proto-ss-rRNA) could have all emerged due to the advantage to save the replication cost of RNA templates for corresponding peptides. At this point, the manuscript should not only have a sense making "very little progress towards a plausible solution".

Moreover, to me, key aspects of Wentao Ma's hypothesis remain rather obscure. The cornerstone of the discussion seems to be the "replication parsimony principle" that is introduced as follows: "Here, by "replication parsimony" I mean that informational sequences in genetic molecules have a tendency to be utilized in a parsimony way under selection pressure, due to the cost involved in their replication." At this point, I am lost as to the meaning of this definition. The subsequent text seems to suggest that the "replication parsimony" principle is a combination of the minimization of replication cost in terms of energy and supply expenditure, and avoidance of the "error catastrophe" by minimizing the genome size. If so, these are well known consideration, and there is no need to introduce a new "principle".

**Author's response: **Yes, the reviewer's understanding on the meaning of "replication parsimony" in this hypothesis is quite right. These may be well known considerations, but no previous hypotheses have applied these considerations to address the problem of the origin of translation. In this context, the replication cost or the room within the size-limited genome which could be saved would allow the appearance of more and longer functional peptides and, finally, proteins. Then, accompanying the appearance of proteinacious enzymes, the proto-organisms would be more efficient in metabolism and replication, and less limited in the genome size, hence evolving towards modern organisms. Certainly, here I define such a "principle" is mainly for the convenience to mention it in the text, not to introduce a new "principle".

In the main part of the article which is the presentation of the hypothesis, Wentao Ma suggests that the evolution of adaptors (tRNAs in the modern translation systems) could have been driven by the advantage of compact nucleotide sites for interaction between polynucleotides and amino acids. Because of stereochemical reasons, at the stage of Direct RNA Templating (DRT), RNA-amino-acid interactions would require oligonucleotides approximately the size of the aptamers that have been shown by the Yarus lab and others to interact with some cognate amino acids, especially Arg. These experiments suggest that about 10-15 nucleotides would be required. By contrast, the adaptor mechanism allows the use of only 3 nucleotides whereas the adaptors themselves are a relatively small expenditure as they can be recycled.

**Author's response: **Yes, this is one of the two main points that the replication parsimony would make sense in the scenario (see below for the second point).

" In principle", this idea makes sense but there are several gaping holes here. First, the plausibility of DRT is very dubious at best. Appendix 1 attempts to provide some explanations but they effectively amount to previously published arguments for potential roles of amino acids and/or non-coded peptides in evolving primordial systems. These are not bad arguments However, the real problem is that DRT is extremely challenging mechanistically, and no new solutions are offered. I would note that even specific binding of individual amino acids by oligonucleotides that is taken here more or less for granted is a difficult problem as the aptamer experiments are not really compelling, perhaps, with the sole exception of arginine.

**Author's response: **Yes, I agree that the plausibility of DRT is under debate. However, the DRT mechanism is the simplest and most direct mechanism for RNA to synthesize peptides, and is very likely to have been adopted initially in the RNA world. The key issue is, as mentioned by the reviewer, whether the evidence for specific binding of individual amino acids by oligonucleotides is compelling. Now, updated evidence on the aa-aptamers has confirmed the specific binding for some complex amino acids, e.g. Arg, His, Ile, Phe, Trp and Tyr [22]. Remarkably, a recent research based on RNA-protein interactions in the ribosome supported this conclusion [41] (I have added this new reference into the manuscript). Additionally, it was shown that those complex amino acids tend to be related to their anticodons (not codons) significantly, but simple, prebiotic amino acids do not (except Asp). This may be an explanation for why only specific aptamers (with cognate anticodons) for complex amino acids were reported. Furthermore, based on this evidence, a surprising and interesting deduction could be derived in the context of the present hypothesis. Namely, complex, biosynthetic amino acids should have entered the genetic code earlier than simple, prebiotic amino acids, which is opposite to the common sense and the assertion in the coevolution theory on the origin of the genetic code [62-64] (see the section "*The compatibility with theories on the origin of the genetic code*", newly added).

Further, Wentao Ma assigns much importance to the role of C-DRT. As far as I understand, the idea here is that complementary sequences would encode distinct peptides, thus leading to further minimization of energy and resource expenditure under the "replication parsimony principle". To me, this idea is sheer speculation without any support in theory or experiment.

**Author's response: **No, this is a key misunderstanding. The aa-binding site of an adaptor would be the same as the corresponding site on the DRT (Figure 2a, b), thus binding the same amino acid. This amino acid would be charged onto the chain end of the adaptor (Figure 2c, d). Therefore, by these adaptors, a C-DRT would "encode" the same peptide (Figure 2e) as its corresponding DRT does (Figure 1b). Because the C-DRT is a necessary part in the replication of the DRT, for a definite number of copies of the functional peptide, fewer turns of the replication would be needed. For example, let us assume that in the lifespan of a DRT molecule, it could synthesize three corresponding peptide molecules, but twelve molecules of the peptide are needed. Originally, four replication turns (one turn would make one C-DRT molecule and then one DRT molecule) would be required to synthesize four DRT molecules, which could match the need. Now, however, only two turns would be required because two DRT molecules and two C-DRT molecules would just match the need (assuming that a C-DRT molecule could also direct the synthesis of three molecules of the peptide in its lifespan). In other words, the introduction of the adaptor-C-DRT mechanism would save ~50% cost in the synthesis of RNA templates for all peptides that were functioning in protocells. The adaptors themselves would bring along some cost, but being cost-efficient because they could be "recycled". This is just the other of the two main points that the replication parsimony would make sense in the scenario (see above for the first point). Namely, the principle of replication parsimony would also account for the direct advantage of the initial introduction of adaptors into the peptide synthesis, as well as that of the subsequent emergence of proto-mRNAs and the proto-ss-rRNA. The corresponding explanation in the text has been strengthened to avoid such a misunderstanding to this key point in the present hypothesis.

Third, more on the technical side, it is of course fine to propose that sites for amino acid binding in the DRT model would be large but I believe this could be made more concrete with actual (even if crude) stereochemical models.

**Author's response: **Yes, but I think the stereochemical model shown in Figure 1a and 1b is just a crude one for this sake. Perhaps a more detailed model is yet unavailable due to our ignorance on the actual structure of a possible DRT.

Fourth, the current article does not even attempt to address the origin of the ribosome whereas to me any discussion of the origin of translation that does not consider ribosomes is woefully incomplete.

**Author's response: **In this hypothesis, the origin of proto-rRNAs, including the proto-ss-rRNA and the proto-ls-rRNA(s), has been discussed. I think that the reviewer meant that the original source of the proto-rRNAs is not addressed. The reason is that I believe that the proto-rRNAs could have derived from ordinary ribozymes acting in the RNA world, considering that both the proto-ls-rRNA(s) and proto-ss-rRNA could have been small functional RNAs with a sequence length around one hundred nucleotides or even less [44,45] (see the section "*Towards RNA-based translation: the origin of proto-rRNAs*"). Unlike proto-tRNAs, which should be introduced "as a set" (being multiple both in number and species), the proto-rRNAs "stand alone" in the proto-translation system, and the necessary to explain their original source is not so apparent (see the section "*A comparison with other hypotheses on the origin of translation*", newly added; see also my responses to reviewer 2).

Wentao Ma cites the previous publications on the origin of translation, in particular Szathmary's coenzyme handle hypothesis [15,30] and the later paper by Wolf and Koonin [16] that extends this hypothesis and puts it in a broader context. These papers suffer from many of the same problems that plague the present article by Wentao Ma. In my opinion, we have to face the fact that we lack an adequate framework for understanding the origin of translation, hence any attempt on detailed modeling is fraught with speculation. Having said this, I believe that these previous publications present both more plausible ideas on the origin of translation and, importantly, deeper critical examination of the problem and potential solutions than the present paper.

**Author's response: **As Crick et al. stated several decades ago, "the origin of protein synthesis is a notoriously difficult problem" [1], now the problem is still notoriously difficult. Even a comparison between those related hypotheses is not a easy job. However, according to the opinion of this reviewer and reviewer 3, I have tried to do such a comparison in the newly added section --- "*A comparison with other hypotheses on the origin of translation*". Overall, I believe that the present hypothesis has given us a chance to overlook the whole scenario, at least, from a novel angle. Concerning the plausibility of the idea in this hypothesis, it has recently been demonstrated by computer simulation the triplet genetic code could have evolved from larger size codes, driven by the selective pressure of saving "resources and time" in replication [39], which just corresponds to one scene in the present scenario (Figure 3, from a to b) (I have cited this new reference in the part "Testing the hypothesis").

**Reviewer 2: **Juergen Brosius, University of Muenster, Muenster, Germany

This is yet another attempt reconstruct the events leading from "so simple beginnings" as a few RNA molecules to a complicated machinery such as the extant translation apparatus. The author correctly stresses multiple times that evolution has no foresight, but at times falls - as we all do - into that "anthropomorphic" trap. In any event, the scenario still leaves large conceptual gaps, such as the question as to which functional RNAs were the precursors of ribosomal RNAs by exaptation. They could not have arisen out of the blue for the purpose of polypeptide synthesis (foresight, see above). The author proposes self-splicing introns as aminoacylation enzymes, but fails to mention self-splicing introns as possible precursors of rRNAs [57]

Other alternatives should be discussed or at least mentioned, such as rRNA evolving from an amino acid tagged RNA that also served as a template *in cis*, reminiscent of the extant tmRNA [50]. Alternatively, I suggested a ribozymic precursor of a synthesase as the ancestor of the primordial ribosome in one of the reviewer comments to the following publication [73].

**Author's response: **Yes, in this hypothesis, I have not answered directly about the question about the precursors of ribosomal RNAs (this point has also been criticized by reviewer 1). The reason is that I believe that the proto-rRNAs could have derived from ordinary ribozymes acting in the RNA world, considering that they might have originally included only one hundred nucleotides or even less [44,45] (see the section "*Towards RNA-based translation: the origin of proto-rRNAs*" and the newly added section "*A comparison with other hypotheses on the origin of translation*"; also see my response to reviewer 1). If there must be a tendency, I would like to agree the viewpoint mentioned by this reviewer -- self-splicing introns as possible precursors of rRNAs [57] (see the last paragraph in Appendix 3). Additionally, I have included the "tmRNA" hypothesis [50] in the section "*A comparison with other hypotheses on the origin of translation*" (Hp10 in Table 1). The paper [73] has discussed some issues relevant to the present hypothesis (see the places where I cite it), but the relationship between the aa-tRNA synthesase ribozymes (r-AARSs) mentioned in it and the ancestor of the primordial ribosome seems unclear.

The moonlighting of non-protein coding RNAs as messenger RNAs in the fledging RNP world has been discussed before [87]. What else should have mRNAs arisen from but other RNAs?

**Author's response: **Yes, the original source of mRNAs has been discussed in some hypotheses (e.g. in Table 1, Hp 1, 4, 5, 10-14), however, the discussions were at best only in an unclear or abstract way. The discussion in [87] was also in such a way. On the contrary, the related discussion in the present hypothesis is clearer. Wherein, it is described that proto-mRNAs would have derived from C-DRTs (Figure 2e to Figure 3a), in the evolving process driven by replication parsimony.

At the beginning of the manuscript, under the heading "Emergence of adapters: the origin of proto-tRNAs and proto-mRNAs", the author discredits amino acids covalently bound to nucleic acids (the RNA genomic tag and coding coenzyme handles hypothesis [15,28-30]) in favor of the direct-RNA-template theory (DRT). Later on, while developing his concept (e.g., see conclusion), the author heavily relies on tRNA with covalently bound amino acids.

**Author's response: **No, apparently, tRNAs with covalently bound amino acids would emerge in the translation system sooner or later. I do not mean to exclude "amino acids covalently bound to nucleic acids", instead, to suppose that the synthesis of initial peptides should have relied on the DRT mechanism and developed into this way only later.

In any event, the DRT model is problematic as discussed previously by Wolf and Koonin in reference [16] and would have ended in a dead end street. The author tried to overcome this problem, but did not convince, in my opinion. In addition, the further developments of the DRT scenarios are problematic. For example, in appendix 1, the concatenation of several amino acid binding sites versus individual ones, does not significantly reduce the replication burden. Also, when the spacer sequences are incorporated as suggested in Figure 1b, the improvement of short peptide synthesis must outweigh the replication disadvantage.

**Author's response: **Yes, I admit that the DRT model is under debate (see also my responses to reviewer 1). However, it is attractive on at least two points: first, it is based on available evidence from aa-aptamer experiments; second, it is the most simple and direct mechanism for RNA to synthesize peptides, which was very likely to be adopted initially in the RNA world, considering that evolution "has no foresight". On the possible route towards DRTs described in Appendix 1, the appearance of concatenation of multiple amino acid binding sites was not attributed to the reduction of the replication cost, but to the efficiency of the replication. Surely, the replication parsimony could not be the only driving force in evolution (see also my responses to reviewer 3). In fact, the incorporation of spacer sequences would even increase the replication cost, but this disadvantage could have been outweighed by the improvement of replication efficiency for these aa-binding sites (i.e. one turn of replication for a number of such sites). Further, as the reviewer mentioned, the advantage of short peptides synthesized on the RNAs with multiple aa-binding sites would strengthen the advantage of this organizational form of multiple aa-binding sites.

A significant problem is the transition from the non-covalent aa-binding pocket to the covalent tag or handle position (Figure 1, from b to c; Figure 2d). Clearly, the idea of self-splicing introns serving as amino acid synthetases is attractive, in part because it can work modularly on all tRNAs without significantly inflating genome size. However, improvement of translation from DRT to covalent binding is more difficult as, in my opinion, DRT is not feasible (see above) and hence this transition could not have been made in connection with translation. What could have been the underlying pressure for shifting to the covalent adducts of RNA and amino acids, without implying foresight and planning for translation? Once more, we are at a point where the related tag and handle hypotheses by Weiner and Szathmáry, respectively, are more parsimonious.

**Author's response: **Yes, this problem is significant. The present hypothesis addresses the problem by supposing the self-aminoacylation of aa-aptamers and then extending this idea by supposing a self-splicing intron as *in cis *aa-tRNA synthetase (Appendix 3). Indeed, as the reviewer pointed out, the attraction of this scene is that the intron can work modularly on all tRNAs without significantly inflating genome size, which is consistent with the principle of replication parsimony. Additionally, it avoids the assumption of a separate set of aminoacyl-tRNA synthetase ribozymes (so called "r-AARSs" in [73] and related papers along this line). The latter should further explain the formation of the specificity between amino acids and the ribozymes, and that between the ribozymes and cognate tRNAs, which would be more complicated to emerge, considering that "evolution can not work like an engineer".

I do not agree that DRT is not feasible, especially considering new evidence coming from aa-aptamers [22] and RNA-protein interactions [41] (see my responses to reviewer 1). In addition, to make the scenario more convincing on the "translation from DRT to covalent binding" (considering the myopia of evolution), I add a new section in Appendices (Appendix 2) to address the problem concerning the original source of adaptors. Therein, it is explained that adaptors might also have come from single aa-aptamers for the retention of amino acids in protocells, as well as "from the degradation or partial replication of DRTs". This alternative scene is also compatible with other scenes in the scenario described in the present hypothesis.

A codon anticodon interaction exceeding much more than 3 base pairs is problematic as the reversibility of the binding will be highly inefficient and, at best, significantly hamper the speed of elongation. In general, the issue of elongation is ignored in the hypothesis presented. The reduction of base pairs between a proto-tRNA and a proto-mRNA also counters the DRT hypothesis, if there is a significant reduction, why do amino acids apparently still bind to anticodons? In appendix 3, the author fails to explain what functions the group I introns might have had prior to their exaptation as temporary parts of proto-tRNAs.

**Author's response: **This problem should be considered in the reverse angle. As it was suggested by Crick et.al. [1], a proto-translation system before the emergence of proto-rRNAs might have adopted more than three base pairs between proto-tRNAs and proto-mRNAs to ensure a stable interaction between them (see also [39]). The 3- base-pair interaction is likely to be too weak [38]. Base pairs of about 5-7 might be suitable, namely, sufficient to ensure the binding of proto-tRNAs while not too tight to prevent their drop. The explanation of "elongation" has been represented in Figure 2e and Figure 3 as the delivery of amino acids between proto-tRNAs. Of course, more efficient elongation should involve the translocation of proto-rRNAs, an issue that is not addressed in the present hypothesis. As the reviewer has noticed, an unavoidable problem is that the reduction of base pairs between a proto-tRNA and a proto-mRNA should be consistent with the binding of amino acids on proto-tRNAs at their self-aminoacylation state. However, Appendix 3 has just been aimed at this problem (Figure 4). To be clearer, I have made a more detailed explanation. As to the possible function of the group I introns prior to their exaptation as temporary parts of proto-tRNAs, I tend to think that by self-inserting and self-splicing, they might have previously been parasitic RNA species, instead of functional elements in RNA-based protocells.

How is the scheme in Figure 4 in agreement with [73]?

**Author's response: **The operational code determines the aa-tRNA specificity nowadays for some amino acids. This fact has brought some speculation that it might also have had some role in the initial formation of aa-tRNA specificity during the origin of proto-translation system in the RNA world [73]. Especially, there have been quite a few studies (from the Rodin's group, the Schimmel's group and others) on the relation of the operational code and the genetic code, which might be able to provide some clues for the evolution of proto-tRNAs' structure because the two codes reside at two spatially opposite end of a tRNA. However, such studies have, at best, returned very obscure results. Indeed, as shown in Figure 4, the 3' strand of the acceptor stem of a proto-tRNA, where the operational code resides, can be brought adjacently to the place of the anticodon loop, which implied that the two codes might have "cooperated" to ensure the specificity of the aminoacylation of the proto-tRNA. However, in the viewpoint of the present hypothesis, the specificity should have been ensured alone by the aa-binding site at the place of the anticodon loop. Therefore, what the approaching of the accept stem and the anticodon loop described in Figure 4 implies is actually that the operational code might have occurred when the proteins (peptides) aiding the group I intron's self-splicing began to substitute the role of the group I intron, evolving towards proteinacious aminoacyl-tRNA synthetases (p-AARSs) (see Appendix 3). In other words, the operational code should have had nothing to do with the initial formation of aa-tRNA specificity during the origin of proto-translation system in the RNA world.

Additionally, by contrast with the scene in Figure 4, which suggest the group I intron as an *in cis *aminoacyl-tRNA synthetase ribozyme, the discussion in [73] assumed a separate set of aminoacyl-tRNA synthetase ribozymes (so called "r-AARSs", acting *in trans*). The latter, as I have mentioned above, should explain both the formation of the specificity between amino acids and the ribozymes, and that between the ribozymes and cognate tRNAs. This "separate assignment" of the aa-tRNA specificity is (much) more complicated, thus (much) less likely to have occurred in the RNA world, considering that "evolution can not work like an engineer". Again, I suggest that the "separate assignment" of the aa-tRNA specificity should have occurred after (or accompanying that) the role of the group I introns was substituted by p-AARSs.

**Reviewer 3: **Arcady Mushegian, Stowers Institute, Kansas City, USA

The related questions of the origin of translation and the origin of genetic code have been examined from various angles for decades, with the seminal book by C. Woese appearing as early as 1968 and the power of comparative genomics applied to the problems in the 21st century. There is so much said and achieved that even the review of the existing theories, their strengths and weaknesses would take more than a brief review. In the pages of Biology Direct, the study of Wolf and Koonin [16] that the author cites, makes a reasonable effort to provide a summary of main theories, and I would also recommend the classification of various theories given by Rob Knight in the published review accompanying this same paper. Just one more of recent independent contribution is Yakhnin AV [49].

**Author's response: **Yes, I agree with the reviewer on that "there is so much said and achieved that even the review of the existing theories, their strengths and weaknesses would take more than a brief review." For a quick start to introduce the main idea of the present hypothesis, I did not comment on various previous hypotheses in the part of "Background", except the DRT hypothesis, which is tightly related with the scenario described here. In the part "The presentation of the hypothesis" where the key issue, the origin of adaptors, was addressed, I discussed the strengths and weaknesses of another two popular hypotheses, the GT (Genomic Tag) and CCH (Coding Coenzyme Handles). According to the opinions of this reviewer (see below) and reviewer 1, in the part "Implication of the hypothesis", I have extended the discussion to some other representative hypotheses (the section "*A comparison with other hypotheses on the origin of translation*"). Among these hypotheses, the Hp5 [16] and Hp14 [49] (Table 1), mentioned above by the reviewer, are the most impressive two on their efforts to describe the whole scenario in detailed intermediate steps, considering the "myopia of evolution". In addition, related theories on the origin of the genetic code has also been discussed (the section "*The compatibility with theories on the origin of the genetic code*"), and a surprising deduction is reached --- complex, biosynthetic amino acids should have entered the genetic code earlier than simple, prebiotic amino acids, which is opposite to the common sense and the assertion in the coevolution theory [62-64].

To this wealth of knowledge and insight, the authors adds a valid consideration of steric hindrances within some of the simplest amino-acid-ligating ribozymes, and suggests that some of the intermediate steps in the evolution of translation may have involved the selection of RNA spacers to overcome this problem. If this is a new idea or a new version of an old idea, it is still, in my view, a minor contribution to the topic.

**Author's response: **Yes, this is an important, new idea associated with "what a DRT should be like". It underscores the replication burden to synthesize functional peptides by the DRT mechanism. However, the most important idea in the present hypothesis is not this, but the one concerning the subsequent evolving process towards a proto-translation system, in which the principle of "replication parsimony" would work.

The author dismisses the corpus of previous work rather cavalierly. Instead, the author should state clearly which of the existing theories of the origin of translation the current proposal is most compatible with and what does it add to our understanding that we did not know before.

**Author's response: **Yes, I agree with the reviewer. Though there have been a lot of previous theories and a comprehensive comparison between them and the present hypothesis is not an easy job, an effort towards this direction is still needed to make the discussion "useful" (this is also the opinion of reviewer 1). Therefore, as mentioned above, I have added the section "*A comparison with other hypotheses on the origin of translation*" and the section "*The compatibility with theories on the origin of the genetic code*".

"Replication parsimony" is in my view an unfortunate expression: "parsimony" is a preference for an explanation that is "least complex", usually understood as "consisting of the smallest number of distinct events". The "replication parsimony" discussed by the author is more along the lines of minimizing the replicon size. Moreover, I am not sure why this would be a major evolutionary force at the early stages of emergence of translation, when counterbalanced by the functional needs.

**Author's response: **Yes, the "replication parsimony" discussed here means "minimizing the size of RNA templates for the synthesis of corresponding peptides", to save replication cost of these templates. Perhaps "replication economy" is a better expression, but I am not quite sure because "economy" has a lot of other meanings. So I have kept the word "parsimony". Surely, the "replication parsimony" is not the only driving force in evolution. Even more, it should not be the major driving force in evolution because it would be counterbalanced by the functional needs (just as the reviewer said), which is just the reason why the life system and its genome could have been becoming more and more complicated. However, when the functional needs (for the first peptides) could be realized by a mechanism (the proto-translation system) that significant lessened the corresponding replication needs (comparing with the DRT mechanism), the principle of "replication parsimony" would work (see also reviewer 1's comment on this principle and my response). More explicitly, the replication cost (or the room within the size-limited genome in the RNA world [24,25]) which could be saved would allow the appearance of more and longer functional peptides and, finally, proteins. This point is just the cornerstone of the present hypothesis.
